# Assessment of migration regularity of phthalates from food packaging materials

**DOI:** 10.1002/fsn3.1863

**Published:** 2020-09-12

**Authors:** Jing‐Min Liu, Chun‐Yang Li, Fei‐er Yang, Ning Zhao, Shi‐Wen Lv, Ji‐Chao Liu, Li‐Jun Chen, Ze He, Yan Zhang, Shuo Wang

**Affiliations:** ^1^ Tianjin Key Laboratory of Food Science and Health School of Medicine Nankai University Tianjin China; ^2^ Beijing San Yuan foods co., LTD. Beijing China; ^3^ Key Laboratory of Food Nutrition and Safety Ministry of Education Tianjin University of Science and Technology Tianjin China

**Keywords:** kinetic analysis, migration, phthalate acid esters, simulation experiment

## Abstract

Phthalate acid esters (PAEs) are one of the essential plastic additives which may lead to plenty of harmful effects, including reproductive toxicity, teratogenicity, and carcinogenicity. Increasing attention has been paid to the migration of plasticizer. In this article, the disposable plastic lunch boxes were taken as the research object. The result showed that dibutyl phthalate (DBP) and diisobutyl phthalate (DIBP) have been mainly found, whose content was 1.5 mg/kg and 2.4 mg/kg, respectively. The LOD was 2 ng/g, and LOQ was 6.7 ng/g. We further investigated the migration of PAEs into the simulated liquid at different temperature conditions. Then, the linear fitting performing by first‐order kinetic migration model revealed that the lower the polarity of the simulated liquid, the larger the rate constant *K*_1_ and initial release rate *V*_0_. The higher the temperature, the bigger the *K*_1_ and *V*_0_.

## Graphical abstract

This study takes disposable plastic lunch boxes as the research object, and determines the types and contents of PAEs, which can provide some data support when considering the safety of lunch boxes. Secondly, polypropylene‐based lunch box was chosen as the migration material. The migration laws of different types of simulants at various temperatures and time points are studied, which lays a foundation for further studying the migration laws of PAEs in food processing and preservation.

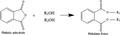

## 1 INTRODUCTION

In recent years, plasticizers have been commonly used in industrial production because they could enhance the flexibility and improve the properties of plastics (Gao, Zhu, & Luo, 2018; Hubinger, 2010). However, misusing them has caused plenty of food safety accidents frequently, which gives rise to consumer concerns and huge losses to society and enterprises (Kawamura, Ogawa, & Mutsuga, 2017; Mgaya‐Kilima, Remberg, Chove, & Wicklund, 2015).

**Figure 1:**
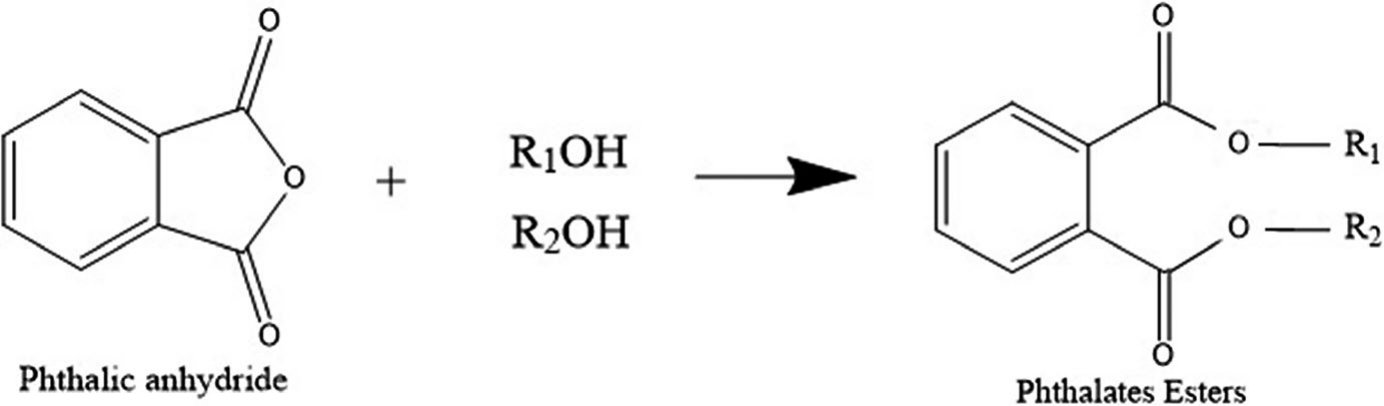
The reaction of synthesizing phthalate acid esters

Phthalate acid esters (PAEs) are one of the main types of plasticizers that obtained by esterification of phthalic anhydride and an alcohol (Figure 1). It is a general term for a wide range of chemical compounds, such as benzyl butyl phthalate (BBP), dibutyl phthalate (DBP), dimethyl phthalate (DMP), di‐2‐(ethylhexyl) phthalate (DEHP), and diisobutyl phthalate (DIBP). Most of them are able to transform hard polyvinyl chloride resins into pliable and processable plastics (Luo et al., 2018). PAEs are small molecules that only bound to plastic matrix by hydrogen bond or van der Waals force. That is why they may migrate from packaging materials especially in oil‐ or fat‐containing food (Kiani et al., 2018; Net, Sempere, Delmont, Paluselli, & Ouddane, 2015; Rastkari, Jeddi, Yunesian, & Ahmadkhaniha, 2018). Although PAEs have no obvious acute toxicity in toxicological experiments, long‐term accumulation will damage the internal organs of the body, interfere with the secretion of human hormones, weaken the fertility, and also have potential carcinogenic effects (Min, Liu, Yang, & Chen, 2014; Wang, Zhang, & Wang, 2015). In particular, the characteristic that can be enriched through the food cycle has been a hot topic in the field related into public health and ecosystem protection (Chen et al., 2014; Farooqi, Rajendran, & Khanam, 2018; Wang et al., 2015). As a consequence, more and more regulatory organizations have limited some PAEs as priority hazardous substances for stringent control. The FDA lists four migration limits on the five PAEs with potential or evident endocrine‐disrupting effect (Du, Ma, Qiao, Lu, & Xiao, 2016). Meanwhile, European Union gives a standard for the migration limits of seven PAEs such as DAP (Diallyl phthalate), DBP, and DEHP (D'Alessandro, Nemkov, & Hansen, 2016).

Several reported pretreatment technique of PAEs is mature relatively, such as liquid–liquid extraction(Lopez‐Feria, Lucena, Cardenas, & Valcarcel, 2009), solid‐phase extraction (Qiao, Wang, Yan, & Yang, 2014), supercritical fluid extraction (Li et al., 2018), solid‐phase microextraction (Li, Su, Li, Sun, & Zhang, 2013), ultrasonic extraction (Fernandez‐Gonzalez, Moscoso‐Perez, Muniategui‐Lorenzo, Lopez‐Mahia, & Prada‐Rodriguez, 2017), microwave extraction (Gao et al., 2018), and accelerated solvent extraction (ASE) method (Armstrong, Rice, Ramirez, & Torrents, 2018). Among which, the principle of ASE is to extract the target through high temperature and high pressure. PAE extraction process has been correlated with temperature, pressure, extraction time, number of cycles, and nitrogen purge volume, etc. Compared with the traditional method, this method shows remarkable efficiency and convenient operation especially for the extraction of esters with solid matrix (Khosravi & Price, 2015). Moreover, ASE has evoked considerable interest in various fields thanks to the extraction solvent is low in consumption and less in time (Richter, Jones, Ezzell, & Porter, 1996).

For the detection technology of PAEs, gas chromatography‐mass spectrometry (GC‐MS) is mainly used, while other methods such as high‐performance liquid chromatography (HPLC) (Ramirez‐Lopez & DeWitt, 2014), liquid chromatography‐mass spectrometry (LC‐MS) (Lambertini et al., 2014), fluorescence detection (Cui et al., 2015) are gradually driving to maturity. Due to possible injury of PAEs on human health, lots of researches had carried about their migration and assessment of potential risks. For example, Oliveira et al. (Oliveira, de Souza, Padula, & Godoy, 2017) developed a mixture design extraction method to evaluate the migration of PAEs in baby bottle. Albert et al. (Guart, Bono‐Blay, Borrell, & Lacorte, 2014) studied the effects of different plastics on the migration of PAEs during the storage of bottled water in Spain, whereas, due to the considerable differences in food matrices, the variety of packaging materials, and the complicated migration process, the development of migration experiment has been greatly restricted.

According to the situation above, some software or mathematical models were used to simplify or even replace a part of migration experiments (Liang, Bi, Wang, & Xu, 2019; Phitakwinai, Thepa, & Nilnont, 2019). A majority of established models are based on Fick's second law of diffusion, which states that the migration occurring in the thickness of a material can be described by a one‐dimensional second‐order partial differential equation. Seliverstov et al. (Seliverstov, 2016) using the second law of diffusion and the Crank model studied the two‐layer model of the single‐layer migration model and established the Begley‐Holhfield model.

In this study, mathematical, computer knowledge, and instrumental detection were combined to make a more accurate and comprehensive assessment of the migration of PAEs in food packaging. The extraction method in plastic lunch boxes that combined with GC‐MS was first built. Furthermore, we studied the migration regularity of DIBP and DBP in four‐simulation solution under different temperature. The results showed that the influence of simulation was significant, and the immersion temperature was proportional to the migration. Finally, the linear fitting results were obtained based on the migration amounts of targets and first‐order kinetic migration model.

## 2 MATERIALS AND METHODS

### 2.1 Chemicals and instrumentation

All chemicals used are analytical grade or higher. Ultrapure water (18.2 MΩcm) obtained from Milli‐Q Ultrapure Water (Millipore) was used throughout. Sixteen phthalate standards: dimethyl phthalate (DMP, 99.0%), diethyl phthalate (DEP, 99.0%), diisobutyl phthalate (DIBP), 99.0%), phthalic acid phthalate (DBP, 99.0%), bis(2‐methoxy) phthalate (DMEP, 99.0%), phthalic acid (4‐A) Benzyl‐2‐pentyl)ester (BMPP, 99.0%), di(2‐ethoxy)ethyl phthalate (DEEP, 99.0%), diamyl phthalate (DPP, 99.0%), Dihexyl phthalate (DHXP, 99.0%), butyl benzyl phthalate (BBP, 99.0%), bis(2‐butoxy) phthalate (DBEP, 99.0%), Dicyclohexyl phthalate (DCHP, 99.0%), di(2‐ethyl)hexyl phthalate (DEHP, 99.0%), diphenyl phthalate (DPHP, 99.0%), Di‐n‐octyl phthalate (DNOP, 99.0%), and dinonyl phthalate (DNP, 99.0%) were purchased from Dr. Ehrenstorfer. Methanol, acetonitrile, absolute ethanol, ethyl acetate, glacial acetic acid, petroleum ether, and ethanol were obtained from Sinopharm Chemical Reagent Co., Ltd. Acetone, potassium dichromate, and concentrated sulfuric acid were purchased from Tianjin Concord Technology Co., Ltd.

The gas chromatography‐mass spectrometry measured was carried out on an Scion TQ triple‐quadrupole gas chromatography‐mass spectrometer equipped a LC‐10AT UV detector (Shimadzu), using a DB‐5 quartz capillary column (30 m × 0.32 mm × 20 μm, Agilent, American).

### 2.2 Extraction of PAEs from lunch boxes

The PAEs in the lunch box are extracted using an accelerated solvent extraction instrument (ASE300). The plastic lunch box is crushed by a mixer; then, 1.0 g (accurate to 1 mg) is weighed accurately and put into a 66‐ml extraction tank with filter paper. At the same time, the static extraction condition is set. After the end of the extraction, the extract in the collection bottle was poured into a 100‐ml round bottom flask and steamed at 40°C to remove the extraction solvent. When the liquid remains about 2 ml, the steaming is stoped, and the remaining solvent is transferred to a 10‐ml glass volumetric flask. The round bottom flask is rinsed for three times with the same extraction solvent and combines the rinse solution into the glass volumetric flask with the extraction solvent. Make up it to 10 ml and shake well. The liquid in the volumetric flask is poured into a glass centrifuge tube and centrifuged at 15,093 *g* for 5 min, and the supernatant is taken for GC‐MS analysis (Detailed parameters see Support Information).

### 2.3 The standard curve of PAEs

A mixed standard solution of DBP and DIBP is prepared at a concentration gradient of 10, 5 and 1 μg/ml, 0.5, 0.1, and 0.05 μg/ml and detected by GC‐MS. Taking the concentration of PAEs as the abscissa and the integrated peak area of the total ion current map as the ordinate, a standard curve is drawn and the linear regression equation is obtained.

### 2.4 The calculation of mobility

According to the data obtained by GC‐MS, the content of each PAEs is calculated, and the calculation formula is as follows:

$$X=\left(6\times C\times V\right)/S$$

*X*: migration amount of the target analyte in mg/kg; *C*: concentration of the target analyte obtained by GC‐MS, in units of mg/L; *V*: volume of the soaking liquid, the unit is L; *S*: contact area of the sample with the migration device, the unit is dm^2^; 6: EU Directive 2004/19/EC specifies 1 mg/kg = 6 mg/dm^2^;

The final migration experiment results are expressed in terms of mobility, that is, the percentage of migration, the ratio of the content of PAEs in the simulated liquid at the end of migration (*A*1, mg/kg) to the content of PAEs in the lunch box (mg/kg):

Mobility=A1/A2

### 2.5 Establishment of migration model

This experiment uses the first‐order kinetic model described by the formula to linearly fit the experimental data obtained from the migration:

$$\ln\left(1‐\frac{M_{t}}{M_{0}}\right)=‐k_{1}t$$

*M_t_*: the migration amount of the PAEs in the simulated liquid after the migration of *t* time; *M_0_*: The content of PAEs in the raw materials of the lunch boxes.

These two data can be directly obtained from the migration experiment and sample content determination. *M_t_*/*M_0_* is the fractional migration of PAEs; *k*_1_ is the first‐order kinetic rate constant; *t* is the migration time. The slope of the linear fitting equation of ln (1 − *M_t_*/*M_0_*) versus *t* gives the kinetic rate constant *k*_1_ of the entire migration process. The above formulas are based on the assumption that the migrations in the simulated liquid are all derived from the raw materials of the lunch box; the PAEs are not lost during the migration from the raw materials to the simulated liquid; the migration process continues uninterrupted. For the model, when the kinetic analysis is performed, if the migration process conforms to the first‐order kinetic migration, the rate constant *k*_1_ of the migration can be directly obtained by the formula, and the initial of the PAEs at *t* = 0 can be further calculated. The release rate *V*_0_ is calculated as follows:

$$V_{0}=M_{0}\times k_{1}$$

## 3 RESULTS AND DISCUSSION

### 3.1 Optimization of extraction conditions

**Figure 2:**
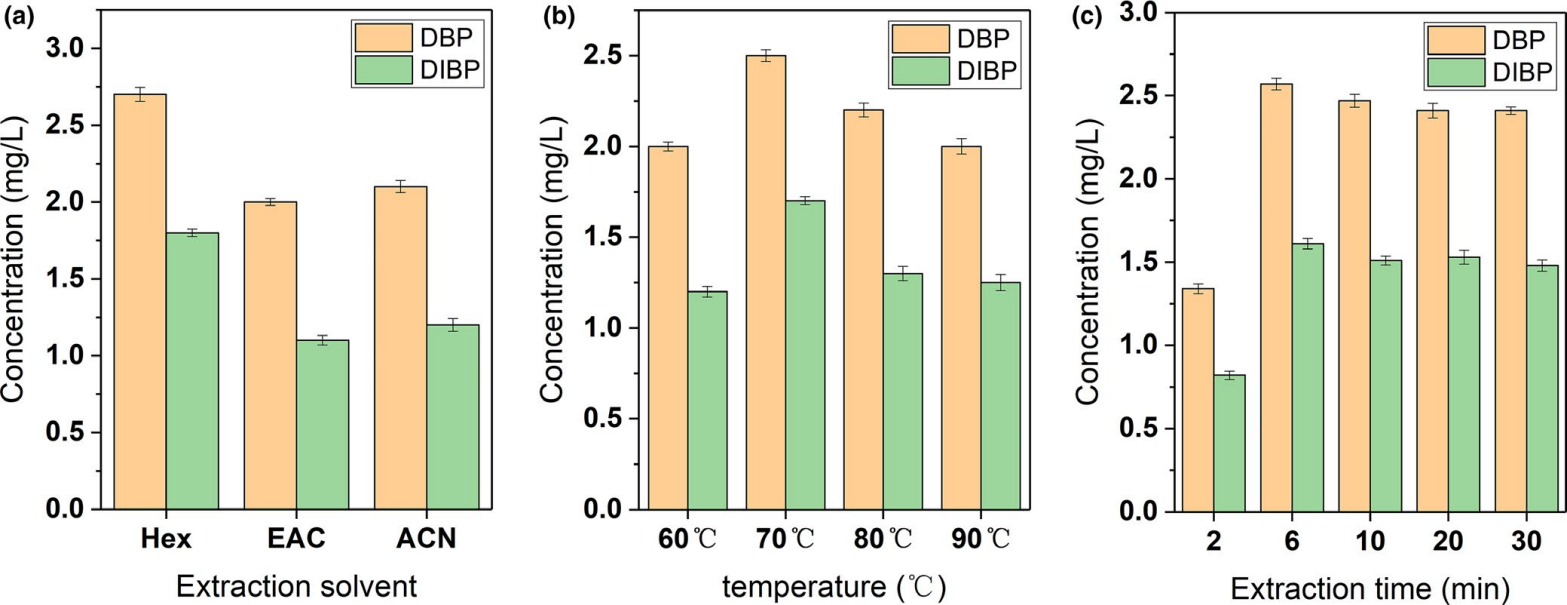
The effects of (A) extraction solvent; (B) extraction temperature; and (C) extraction time for extraction efficiency

Among the three extraction solvents for extracting PAEs, the extraction efficiency of n‐hexane is significantly higher than that of ethyl acetate and acetonitrile (Figure 2a). The content of DIBP and DBP reached 1.6 mg/kg and 2.7 mg/kg, respectively. As is known to all, n‐hexane has the weakest polarity compared with the other two solvents. Due to the principle of similar compatibility, DIBP and DBP have higher solubility in n‐hexane. Next, during the optimization of the extraction temperature, the efficiency reaches a maximum at 70°C and then decreases as the temperature rises again (Figure 2b). It is because that ASE is pressurized heating extraction, the targets cannot be completely separated from lunch boxes under at lower temperatures. After that, the molecular thermal motion of PAEs is accelerated, which causes a increased target content. However, the stability of PAEs will decrease after heating continuously, and thus, the extraction content is also decreased. At the same time, the plastic will soften and shrink if the temperature is too high, the surface area in contact with the extraction solvent will be reduced to affect the extraction efficiency. As for the extraction time, it can be seen from Figure 2c that when the extraction time is 6 min, the content of PAEs is the largest. Afterward, it decreased slightly with the prolongation of extraction time. When it is longer than 10 min, the concentration of DIBP and DBP basically stabilized, which is not much different from that at 10 min. Hence, the PAEs are extracted with n‐hexane at 70°C for 6 min.

### 3.2 GC‐MS analysis of PAEs

Under the chromatographic separation conditions, 16 kinds of PAEs can be effectively separated. After the library search on GC‐MS, the retention time and qualitative and quantitative ions of various PAEs were as showed in Table 1:

**Table 1:** The Retention time, qualitative, and quantitative ion of PAEs

Compound Name	CAS Number	RT (min)	Quantitative Ions	Qualitative Ions
Dimethyl phthalate	131‐11‐3	8.94	163	77,135,194
Diethyl phthalate	84‐66‐2	10.01	149	177,121,222
Benzyl phthalate	120‐51‐4	11.36	105	91,77,194,212
Diisobutyl phthalate	84‐69‐5	12.04	149	223,205,167
Dibutyl phthalate	84‐74‐2	12.89	149	223,205,121
Ethylene glycol phthalate	117‐82‐8	13.32	59	149,104,76
2‐Pentyl phthalate	146‐50‐9	14.07	149	85,251,167
Ethoxyethyl phthalate	605‐54‐9	14.48	45	72,149,221
Dipentyl phthalate	131‐18‐0	14.86	149	237,76,104
Dihexyl phthalate	84‐75‐3	17.17	149	104,251,76
Benzyl butyl phthalate	85‐68‐7	17.39	149	91,206,104
Hexylethylhexyl phthalate	75673‐16‐4	18.47	149	70,104,251,176
Dibutoxyethyl phthalate	117‐83‐9	18.87	149	101,85,193
Dicyclohexyl phthalate	84‐61‐7	19.78	149	167,83,249
Ethylhexyl phthalate	117‐81‐7	19.88	149	167,279.71
Dioctyl phthalate	117‐84‐0	22.29	149	279,261,167

### 3.3 Determination of PAEs

**Figure 3:**
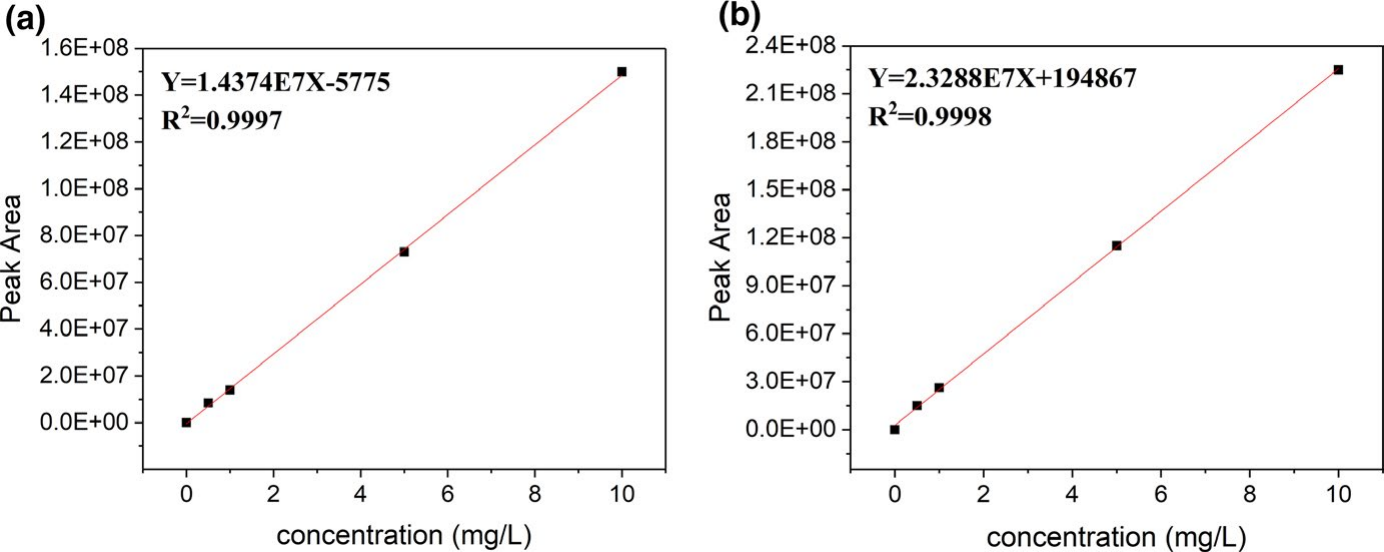
Gas chromatography‐mass spectrometry standard curve of (A) diisobutyl phthalate and (B) dibutyl phthalate

Qualitative analysis of PAEs in the lunch box was carried out by GC‐MS. The results showed that PAEs were detected in all four lunch boxes, mainly DBP and DIBP. It can be seen from Figure 3 that DIBP and DBP have good linearity in the concentration range of 0.05–10 mg/L. The LOD is 2 μg/ml, and LOQ is 6.7 μg/ml.

According to the National standards established by the Ministry of Health of China, the maximum amount of DIBP and DBP added to plastics is 100 mg/kg (China, 2008). In the detection of PAEs in four commercially available lunch boxes (Tables S1–S4), the content of the analytes in the four lunch boxes all meets the standard. Among them, the first type of lunch box with polypropylene (PP) has the highest content of DIBP and DBP comparing the four kinds of lunch boxes, which are 0.53‐1.84 mg/kg and 1.39‐2.83 mg/kg, respectively. Therefore, this kind of lunch box is chosen as the migration experiment material.

### 3.4 Migration of PAEs at different temperatures

Nowadays, many research has been carried out on PAEs migration (Fasano, Bono‐Blay, Cirillo, Montuori, & Lacorte, 2012; Xu et al., 2010; Yang, Xie, Tian, & Yang, 2013), but there is no systematic study in the lunch box. The reasons may be that there are many restrictions on the simulation test. First, the selection of materials is difficult. Plastic containers have a wide variety of materials, and the most common ones are polyvinyl chloride (PVC), polypropylene (PP), polycarbonate (PC), etc. Secondly, the differences in immersion method and migration device will lead to considerable variation of results. According to the conclusion obtained in *3.2*., a PP lunch box with a large content of PAEs and a uniform distribution is selected for simulation experiments. In order to evaluate the safety problems caused by PAEs migration in the daily use of the lunch box, four different temperature conditions are set up to simulate the food storage (4°C), room temperature (25°C), or high temperature (40°C, 60°C).

#### 3.4.1 Migration of PAEs in simulated liquid at 4°C

**Figure 4:**
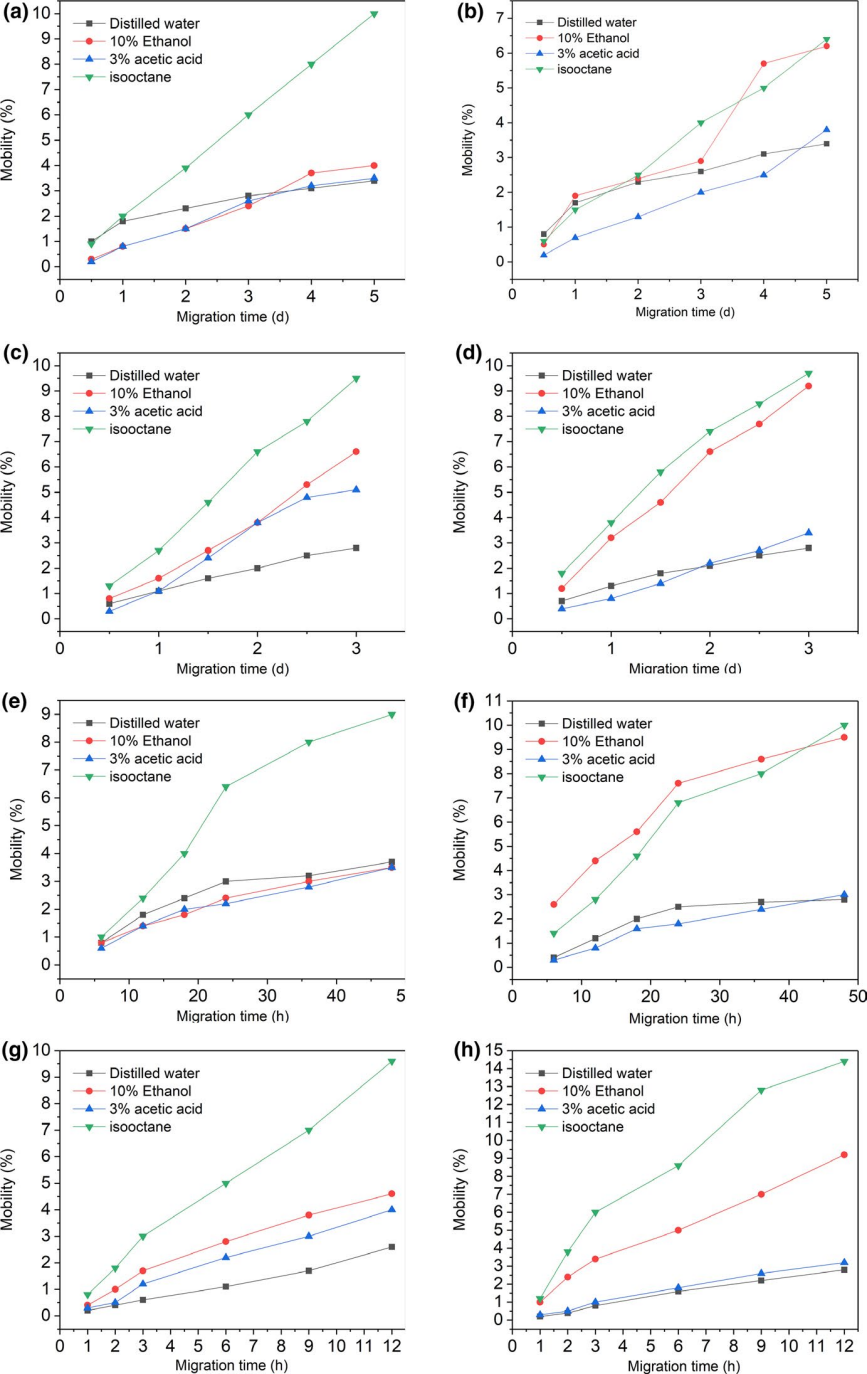
Migration of diisobutyl phthalate, dibutyl phthalate from materials to simulation at 4°C (A, B); 25°C (C, D); 40°C (E, F); 60°C (G, H)

At 4°C, the mobility of DIBP in isooctane is much higher than that of the other three simulated liquids, reaching 9.76% (Figure 4a). The migration rate is the fastest on the third day because the PAEs are a lipophilic substance which is more soluble in isooctane. In distilled water, the migration is not obvious, and it may for the reason that its low solubility in water. The migration of DIBP in 10% ethanol is greater than 3% acetic acid, which consistent with the conclusions drew by Coltro et al. (2014): The migration amount of PAEs in acidic food‐simulating liquid is lower than that of alcohol‐containing food‐simulating liquid. At the beginning, DBP migrates quickly and then decreased slightly (Figure 4b). The amount of it in 10% ethanol changed rapidly from the 0.5th day to the third day, and the rate decreased slightly after the mobility was higher. Within 5 days, the amount of DBP was the highest, reaching 6.42%. Its reason is the same as DIBP.

#### 3.4.2 Migration of PAEs in simulated liquid at 25°C

The mobility of DIBP at 25°C is higher than that at 4°C when the other conditions are same (Figure 4a,c). As the temperature increases, the free energy of the high‐energy molecular segment in PP is sufficient to enhance its mobility because PAEs are a small molecular substance. Then, the segment is free to rotate with the number of conformations is increased, and the shape of the molecular chain changes. Finally, the cavity is formed inside the lunch box, and the free energy obtained by the PAEs is increased by the temperature, so that it can get rid of the interaction force between the PP molecules, and migrate from the polymer successfully (Silva, Freire, Sendon, Franz, & Losada, 2009). In addition, the Arrhenius empirical formula shows that as the temperature rises, the activation energy of small molecules increases accordingly, which is more conducive to material migration. At 25°C, the mobility of DIBP is the highest in isooctane, reaching 9.59%. Meanwhile, the migration of DBP is similar to that of DIBP (Figure 4d). The migration of DBP in the isooctane simulated liquid is higher than that of the other three simulated liquids, reaching 9.94%. Changes in the food matrix play a crucial role in the migration of the polymer, such as the content of oil, the content of ethanol, the shape of the sample, and the content of protein.

#### 3.4.3 Migration of PAEs in simulated liquid at 40°C

It can be seen from Figure 4e that the mobility of DIBP in isooctane is significantly higher than that of the other three simulants at 40°C, reaching 9.12% (48 hr). The lipophilic properties of PAEs are demonstrated once again.

#### 3.4.4 Migration of PAEs in simulated liquid at 60°C

Due to the higher temperature, the migration time is shorter. The results show that the mobility of DIBP at 60°C is higher than that at other lower temperatures (Figure 4a,c,e and g), indicating the temperature has a significant impact on the amount of DIBP migration. Comparing the migration of PAEs in four different simulated liquids, it is found that the migration of isooctane is the highest, and the migration rate is very fast in the early 9 hr. The mobility of DBP at 60°C is higher than that at other lower temperatures, which indicates that the temperature directly affects the migration rate and content of targets in the simulated liquid (Figure 4b,d,f and h).

Based on the above results, it can be concluded that PAEs are more likely to migrate in oily food. Therefore, consumers are advised not to store food in plastic lunch boxes for a long time. In the meantime, it is also necessary to avoid high‐temperature greases in the process of using plastic lunch boxes.

### 3.5 Kinetic analysis of migration in simulated liquid at different temperatures

The first‐order kinetic equations are used to linearly fit the migration data of PAEs. If *R* ^2^ > 0.9, the first‐order kinetic equation can be used to describe the migration process. The kinetic rate constant *K*_1_ can be calculated from the fitting result, and the initial release rate *V*_0_ can be obtained from *K*_1。_

At 4°C, the correlation coefficient value *R* ^2^ of the linear equation obtained by DIBP and DBP fitting is greater than 0.9 (Tables 2 and 3), indicating that the migration of these two PAEs can be described by the first‐order kinetic equation. Since PAEs is a lipophilic substance, isooctane has a stronger affinity with targets, and it is more likely to cause swelling of PP materials and further penetration into the interior. The rate constant and initial release rate in the isooctane are larger, which ultimately affects the migration.

**Table 2:** Kinetic analysis for DIBP migration in four different simulated solutions at 4°C

Simulated liquid	Linear fitting equation	*R* ^2^	*k*_1_	*V*_0_
Distilled water	*Y* = −0.0050*X *− 0.01	0.95202	0.0050	0.00765
3% Acetic acid	*Y* = −0.0084*X* + 0.0005	0.97585	0.0084	0.01285
10% Ethanol	*Y* = −0.0092*X* + 0.0014	0.97782	0.0092	0.01408
Isooctane	*Y* = −0.0210*X* + 0.0014	0.99591	0.0210	0.03213

**Table 3:** Kinetic analysis for DBP migration in four different simulated solutions at 4°C

Simulated liquid	Linear fitting equation	*R* ^2^	*k*_1_	*V*_0_
Distilled water	*Y* = −0.0057*X *− 0.0094	0.94427	0.0057	0.01442
3% Acetic acid	*Y* = −0.0067*X* + 1.8548	0.98736	0.0067	0.01695
10% Ethanol	*Y* = −0.0132*X *− 6.2438	0.99892	0.0132	0.03340
Isooctane	*Y* = −0.0278*X* + 0.0051	0.98663	0.0278	0.07033

The dynamic equations in the simulated liquid have a good linear fit at 25°C, which can be calculated by the first‐order kinetic equation (Tables 4 and 5). At this time, the migration rate constant in the same simulation is higher than 4°C. The polarity of a substance is an important cause of the rate constant. The lower the polarity, the faster the migration.

**Table 4:** Kinetic analysis for DIBP migration in four different simulated solutions at 25°C

Simulated liquid	Linear fitting equation	*R* ^2^	*k*_1_	*V*_0_
Distilled water	*Y* = −0.0092*X *− 0.0027	0.99567	0.0092	0.01417
3% Acetic acid	*Y* = −0.0216*X* + 0.0067	0.96961	0.0216	0.03542
10% Ethanol	*Y* = −0.0376*X* + 0.0101	0.98332	0.0376	0.07806
Isooctane	*Y* = −0.0493*X* + 0.0107	0.99582	0.0493	0.08036

**Table 5:** Kinetic analysis for DBP migration in four different simulated solutions at 25°C

Simulated liquid	Linear fitting equation	*R* ^2^	*k*_1_	*V*_0_
Distilled water	*Y* = −0.0090*X *− 0.003	0.99368	0.0090	0.02277
3% Acetic acid	*Y* = −0.012*X* + 0.0033	0.99901	0.012	0.03036
10% Ethanol	*Y* = −0.047*X* + 0.01	0.98332	0.047	0.11891
Isooctane	*Y* = −0.049*X* + 0.0107	0.99584	0.049	0.12397

Tables 6 and 7 suggest that the first‐order kinetic model can also be used to describe its migration law. Since 3% acetic acid, 10% ethanol, and isooctane are weak in polarity, the affinity between molecules is stronger. During the process of contact between the simulated liquid and the material, the low polarity of the simulated liquid makes it easier to swell the material and further penetrate into the inside of the lunch box, so that the rate constant *K*_1_ and the initial release rate *V*_0_ are larger.

**Table 6:** Kinetic analysis for DIBP migration in four different simulated solutions at 40°C

Simulated liquid	Linear fitting equation	*R* ^2^	*k*_1_	*V*_0_
Distilled water	*Y* = −0.017*X *− 0.0080	0.90499	0.017	0.02788
3% Acetic acid	*Y* = −0.018*X *− 0.005	0.94552	0.018	0.02952
10% Ethanol	*Y* = −0.030*X *− 0.001	0.94446	0.030	0.04920
Isooctane	*Y* = −0.061*X* + 0.0035	0.98694	0.061	0.10004

**Table 7:** Kinetic analysis for DBP migration in four different simulated solutions at 40°C

Simulated liquid	Linear fitting equation	*R* ^2^	*k*_1_	*V*_0_
Distilled water	*Y* = −0.014*X *− 0.004	0.90138	0.014	0.03542
3% Acetic acid	*Y* = −0.018*X *− 6.804	0.96703	0.018	0.04554
10% Ethanol	*Y* = −0.063*X *− 0.0352	0.92147	0.063	0.16167
Isooctane	*Y* = −0.064*X* + 5.7058	0.98660	0.064	0.16192

It can be seen from Tables 8 and 9 that as the polarity of the substance decreases, both *K*_1_ and *V*_0_ increase. Comparing the rate constants of DIBP and DBP at 4, 25, and 40°C, it is found that *K*_1_ is increased with increasing temperature in the same simulated liquid.

**Table 8:** Kinetic analysis for DIBP migration in four different simulated solutions at 60°C

Simulated liquid	Linear fitting equation	*R* ^2^	*k*_1_	*V*_0_
Distilled water	*Y* = −0.0024*X* + 4.45098	0.99403	0.0576	0.09446
3% Acetic acid	*Y* = −0.0037*X *− 2.19608	0.99194	0.0888	0.14563
10% Ethanol	*Y* = −0.0038*X *− 0.0032	0.97679	0.0912	0.14957
Isooctane	*Y* = −0.0084*X *− 0.0016	0.99462	0.2016	0.33062

**Table 9:** Kinetic analysis for DBP migration in four different simulated solutions at 60°C

Simulated liquid	Linear fitting equation	*R* ^2^	*k*_1_	*V*_0_
Distilled water	*Y* = −0.0024*X *− 2.35294	0.99233	0.0576	0.14573
3% Acetic acid	*Y* = −0.0031*X *− 4.21569	0.99189	0.0744	0.18823
10% Ethanol	*Y* = −0.0044*X *− 0.0032	0.99233	0.1056	0.26717
Isooctane	*Y* = −0.0094*X *− 0.0066	0.99076	0.2256	0.57077

In a summary, the type and temperature of the food simulant affect the migration process. The higher the temperature, the greater the free activation energy obtained by small molecules, the more intense the molecular motion inside the substance, and the more likely the migration occurs. The higher the polarity of the simulated liquid, the weaker the affinity of the simulated liquid and the raw material, the harder it is to diffuse into the raw material, the lower the value of *K*_1_ and *V*_0_. *K*_1_ and *V*_0_ directly reflect the migration effect of PAEs in the food simulation.

## 4 CONCLUSIONS

This research takes a disposable plastic lunch box as the research object, and determines the type and content of PAEs, which can provide some data support for consumers when considering the safety of lunch boxes. Secondly, the migration of different kinds of simulants at various temperatures and time is studied using a PP lunch box as the migration material, which lays a foundation for further study on the migration of polymers in food contact materials.

## CONFLICT OF INTEREST

The authors declare no conflict of interest.

## Supporting information

Supplementary Material
