# Supplementary material for: Assessment of migration regularity of phthalates from food packaging materials
Source: Food Sci Nutr. 2020 Sep 12;8(10):5738–47. doi: 10.1002/fsn3.1863 (PMC7590312; doi:10.1002/fsn3.1863)
Supplement: Supplementary file 1 — Supplementary Material [file FSN3-8-5738-s001.docx]

**Supporting Information**

Assessment of Migration Regularity of Phthalates from Food Packaging Materials

Jing-Min Liu^1†^, Chun-Yang Li^1†^, Ning Zhao^1^, Shi-Wen Lv^1^, Zhi-Hao Wang^1^, Ji-Chao Liu^2^, Li-Jun Chen^2^, Ze He^3^, Yan Zhang^1^* and Shuo Wang^1^*

^1^ Tianjin Key Laboratory of Food Science and Health, School of Medicine, Nankai University, Tianjin 300071, China

^2^ Beijing San Yuan foods co., LTD., No. 8 Yingchang Road, Yinghai, Daxing District, Beijing 100076, China

^3^ Key Laboratory of Food Nutrition and Safety, Ministry of Education, Tianjin University of Science and Technology, Tianjin 300457, China

^†^ Jing-Min Liu and Chun-Yang Li should be considered joint first author

***** Correspondence: wangshuo@nankai.edu.cn; Tel.: +86-22-85358445

yzhang@nankai.edu.cn; Tel.: +86-22-85358473

**1 | MIGRATION EXPERIMENT DESIGN**

(1) Selection of raw materials for lunch boxes: Comparing the results of the four kinds of lunch boxes, a lunch box made of polypropylene (PP) with a high phthalate content was selected as the migration experimental material.

(2) Selection of food simulating liquid: Usually we divide food into 4 types: non-acid, acid, alcohol and oil. In combination with the requirements of the US FDA and the European Community EC, the following four simulated fluids were selected for migration experiments: distilled water, 3% acetic acid, 10% ethanol, isooctane.

(3) Selection of migration device and sample immersion: According to the single-sided contact of the raw material of the study with the food simulant, a specific single-sided migration device was selected, and the contact material had an area of ​​6 cm ^2^ and a regular circular shape. Cut the polypropylene (PP) material of the lunch box into a circular shape of the same size as the interface of the migration device, place it on the bottom cover of the migration device, press the sealing ring, and inject 2 mL of the infusion solution per square centimeter of contact material. 12 mL of the food simulating solution was added, and the lid on the top of the device was placed, and the migration device was placed under different temperature conditions for the migration experiment.

(4) Immersion experiment under different temperature conditions: the immersion test materials were immersed in four kinds of simulated liquids by single-sided immersion method to study the phthalate esters in the raw materials of the lunch boxes at different temperatures. The change rule of contact time was used to determine the content of phthalates in the simulated liquid. In this experiment, four temperature conditions were set, which were 4 °C, 25 °C, 40 °C, and 60 °C, respectively. The sampling time interval of the simulated liquid was different under different temperature conditions.

(5) Pretreatment of food simulating liquid:

(i) Pretreatment of distilled water simulating solution: Pour the simulated liquid into a 25 mL stoppered glass centrifuge tube, select n-hexane as the extraction solvent, and add 3 mL of n-hexane according to the ratio of the simulated liquid to the extraction solvent of 4:1. The vortex oscillator was shaken at 2500 r/min for 5 min. After the shaking, the layer was allowed to stand, and the supernatant was transferred to a 10 mL volumetric flask. 3 mL of n-hexane was added to the simulated solution for secondary extraction. The two supernatants were combined and the volume of n-hexane was adjusted to a 10 mL volumetric flask. The volume of the simulated solution was transferred to a glass test tube, blown dry in a nitrogen blower, and accurately resorbed with 2 ml of n-hexane using a 1 ml gas-tight syringe for GC-MS detection.

(ii) Pretreatment of 3% acetic acid simulant: same as distilled water

(iii) Pretreatment of 10% ethanol simulated liquid: same as distilled water

(iv) Pretreatment of isooctane simulant: isooctane is an organic solvent, which is miscible with phthalic acid and can be directly detected by GC-MS.

**2 | GC-MS PARAMETERS**

1. Gas chromatography conditions

| project | parameter |
| --- | --- |
| Inlet temperature | 280 ° C |
| Column heating program | 60 ° C (1min), then raise to 220 ° C at 20 ° C min^-1^ (1min), finally raise to 300 °C at 5 °C min ^-1^ (10min) |
| Carrier gas | High purity helium |
| Flow rate | Flow rate 1ml min ^-1^ |
| Injection volume | 1µl |

(2) Mass spectrometry conditions

| project | parameter |
| --- | --- |
| Ionization mode | High-sensitivity electron bombardment source (EI) |
| Monitoring method | Select ion scanning mode (SIM) |
| Ion source temperature | 230 °C |
| Transmission line temperature | 250 °C |
| Ionization energy | 70 eV |
| Solvent delay | 3 min |
| Sample rinses | 3 times |

**3 | OPTIMIZATION OF EXTRACTION METHODS**

| project | parameter |
| --- | --- |
| extraction pressure | 1500 psi |
| flushing volume | 60% |
| static cycle | twice |
| nitrogen purge time | 120 sec |

Optimization of extraction temperature: 60 ° C, 70 °C, 80 °C, 90 °C and 100 °C;

Optimization of extraction time: the total extraction time is 6 min, 10 min, 20 min, 30 min, 40 min;

Optimization of extraction solvent: n-hexane, ethyl acetate and acetonitrile.

**TABLE S1** | The content of PAEs in first sample.

| Sample number | DIBP content | RSD | DBP content | RSD |
| --- | --- | --- | --- | --- |
|  | **average value±SD（mg/kg）** |  | **average value±SD（mg/kg）** |  |
| 1 | 1.57±0.12 | 7.64% | 2.79±0.14 | 5.01% |
| 2 | 1.84±0.09 | 4.89% | 2.54±0.07 | 2.76% |
| 3 | 0.90±0.05 | 5.56% | 2.83±0.20 | 7.07% |
| 4 | 1.55±0.11 | 7.09% | 1.65±0.11 | 6.67% |
| 5 | 1.27±0.08 | 6.29% | 1.63±0.06 | 3.68% |
| 6 | 0.8±0.05 | 6.25% | 2.24±0.14 | 6.25% |
| 7 | 0.53±0.06 | 11.32% | 1.89±0.07 | 3.70% |
| 8 | 1.11±0.08 | 7.21% | 1.61±0.16 | 9.93% |
| 9 | 1.28±0.02 | 1.56% | 1.39±0.08 | 5.76% |
| 10 | 1.48±0.04 | 2.70% | 2.83±0.09 | 3.2% |

**TABLE S2 |** The content of PAEs in second sample.

| Sample number | DIBP content | | RSD | DBP content | | RSD |
| --- | --- | --- | --- | --- | --- | --- |
|  | **average value±SD（mg/kg）** | |  | **average value±SD（mg/kg）** | |  |
| 1 | 0.7±0.04 | 5.71% | | 1.79±0.12 | 6.70% | |
| 2 | 0.49±0.02 | 4.08% | | 0.93±0.05 | 5.38% | |
| 3 | 0.38±0.01 | 2.63% | | 0.87±0.03 | 3.45% | |
| 4 | 0.05±0.003 | 6.0% | | 1.27±0.03 | 2.36% | |
| 5 | 0.13±0.01 | 7.69% | | 1.52±0.06 | 3.95% | |
| 6 | 0.11±0.01 | 9.09% | | 0.71±0.02 | 2.82% | |
| 7 | 0.09±0.004 | 4.44% | | 0.55±0.01 | 1.82% | |
| 8 | 0.14±0.01 | 7.14% | | 1.54±0.08 | 5.19% | |
| 9 | 0.41±0.02 | 4.88% | | 1.01±0.10 | 9.90% | |
| 10 | 0.29±0.03 | 10.34% | | 0.54±0.04 | 7.41% | |

**TABLE S3 |** The content of PAEs in third sample.

| Sample number | DIBP content | | RSD | DBP content | | RSD |
| --- | --- | --- | --- | --- | --- | --- |
|  | **average value±SD（mg/kg）** | |  | **average value±SD（mg/kg）** | |  |
| 1 | 0.36±0.02 | 5.56% | | 0.53±0.04 | 7.55% | |
| 2 | 0.39±0.03 | 7.69% | | 0.58±0.03 | 5.17% | |
| 3 | 0.19±0.01 | 5.26% | | 0.99±0.06 | 6.06% | |
| 4 | 0.16±0.01 | 6.25% | | 1.29±0.08 | 6.20% | |
| 5 | 0.15±0.01 | 6.67% | | 1.52±0.01 | 0.66% | |
| 6 | 0.09±0.01 | 11.11% | | 1.44±0.01 | 0.69% | |
| 7 | 0.11±0.01 | 9.09% | | 2.46±0.13 | 5.28% | |
| 8 | 0.08±0.01 | 12.50% | | 1.37±0.05 | 3.65% | |
| 9 | 0.20±0.01 | 5.0% | | 0.29±0.01 | 3.45% | |
| 10 | 0.21±0.02 | 9.52% | | 0.32±0.02 | 6.25% | |

**TABLE S4 |** The content of PAEs in forth sample.

| Sample number | DIBP content | | RSD | DBP content | | RSD |
| --- | --- | --- | --- | --- | --- | --- |
|  | **average value ± SD (mg/kg)** | |  | **average value ± SD (mg/kg)** | |  |
| 1 | 0.22±0.02 | 9.09% | | 0.35±0.02 | 5.71% | |
| 2 | 0.16±0.01 | 6.25% | | 0.26±0.02 | 7.69% | |
| 3 | 0.30±0.01 | 3.33% | | 1.34±0.12 | 8.96% | |
| 4 | 0.17±0.008 | 4.71% | | 0.94±0.08 | 8.51% | |
| 5 | 0.12±0.005 | 4.17% | | 1.40±0.11 | 7.86% | |
| 6 | 0.17±0.005 | 2.94% | | 0.95±0.03 | 3.16% | |
| 7 | 0.14±0.002 | 1.43% | | 0.23±0.01 | 4.35% | |
| 8 | 0.11±0.001 | 0.91% | | 0.16±0.009 | 5.63% | |
| 9 | 0.28±0.01 | 3.57% | | 1.11±0.08 | 7.21% | |
| 10 | 0.19±0.01 | 5.26% | | 0.87±0.06 | 6.89% | |
